# Intraoperative complications in total hip arthroplasty using a new cementless femoral implant (SP-CL^®^)

**DOI:** 10.1186/s10195-020-00548-6

**Published:** 2020-05-25

**Authors:** Kaspar Tootsi, Loviisa Lees, Boris Geiko, Aare Märtson

**Affiliations:** 1 Department of Traumatology and Orthopaedics, University of Tartu, Puusepa 8, Tartu, 51014 Estonia; 2 Traumatology and Orthopaedics Clinic, Tartu University Hospital, Puusepa 8, Tartu, 51014 Estonia

**Keywords:** Cementless, Hip arthroplasty, Complications, Intraoperative fracture, SP-CL

## Abstract

**Background:**

Considering the excellent results already achieved in total hip arthroplasty (THA), new implants must be at least as safe as currently used implants and lead to longer survival. A new cementless femoral stem, SP-CL^®^, has been introduced. The aim of this study is to evaluate intraoperative complications and assess the risk factors of THA with the SP-CL^®^ implant.

**Materials and methods:**

All THA patients who were operated on using the SP-CL^®^ (LINK, Hamburg, Germany) implant between 2015 and 2018 were included in the analysis. Data were collected from medical records from national and hospital electronic databases. Radiological measurements were made from standard pre- and postoperative radiographs.

**Results:**

A total of 222 THA were performed using the SP-CL^®^ implant. The average age of the patients was 56 years (14–77 years). There were 1 transient sciatic nerve injury, 1 acetabular fracture, and 11 (5.0%) intraoperative femoral fractures (IFF), of which 7 were treated with cerclage wire or titanium band during the operation while the other fractures were treated conservatively. None of the IFF patients were revised due to fracture during the follow-up period (one revision due to infection). The radiographic morphology of proximal femur was associated with increased risk of IFF (*p* = 0.02).

**Conclusions:**

The results of the current study demonstrate a 5% incidence of IFF when using the LINK SP-CL^®^ femoral stem in THA. The radiographic morphology of the proximal femur was an important predictor of IFF and should be assessed when using SP-CL^®^.

**Level of evidence:**

Level 4.

## Introduction

Total hip arthroplasty (THA) has been claimed to be the operation of the century due to its excellent results [1]. Nevertheless, there are complications that can lead to revision surgery. One of the most problematic groups among the THA population is younger patients, whose lifetime risk of revision is up to 35% compared with 5% for patients aged over 70 years [2]. Since global trends show an increase in THA in this younger age group, it is crucial to avoid complications and improve implant survival [3, 4]. Aseptic loosening is the major reason for revision after THA [5]. The growing use of cementless implants has been shown to improve the survival rate in younger patients [6]. Cementless implants have also increasingly been used among the elderly population, which should not be encouraged because of the higher complication rate [7].

One of the concerns related to the use of cementless implants in THA is the increased risk of intraoperative femoral fracture [8]. In THA using cementless implants, the geometrical fit of the implant is essential to achieve good primary stability. Thus, thorough preparation of the femoral canal and correct sizing and fitting of the implant are of utmost importance to ensure the longevity of the implant.

IFF occur most often at calcar, greater trochanter, and femoral diaphysis [8]. Since IFF lead to poorer functional outcomes and decreased patient satisfaction, it is important to identify the risk group and avoid complications [9].

A novel cementless femoral implant (SP-CL^®^) has been developed, offering several new features that might improve the outcome of THA: (1) grooves in the proximal part of the stem to provide early rotational stability, (2) an anatomic S-shape of the implant that decreases the morphometric mismatch with the proximal femur, (3) a CaP coating on the proximal two-thirds to provides osseointegration for secondary stability, and (4) a polished tip of the implant to allow gliding in the medullary canal, which does not cause stress risers and might decrease the incidence of thigh pain.

The aim of the current study is to analyze the intraoperative complications and assess the risk factors for IFF in THA using the SP-CL^®^ implant.

## Materials and methods

All THA patients who were operated using the SP-CL^®^ (LINK, Hamburg, Germany) CaP-coated implant at Tartu University Hospital between 2015 and 2018 were included in this study. The data were collected retrospectively from local hospital records and from the national electronic medical database. The medical center has previous experience with Zweymüller and uncemented Müller-type implants from different companies since the year 1997. The decision of whether to use the SP-CL^®^ implant was made by the operating orthopedic surgeon based on the patient’s age, fragility, bone morphology, comorbidities, and activity level. Posterolateral and direct lateral surgical approaches were used with the patient in lateral decubitus position with a cushion between the legs.

Data for sex, age, weight, height, and comorbidities were obtained from medical records. IFF data were collected from medical records and by evaluation of radiographs.

### Radiological assessment

**Fig. 1:**
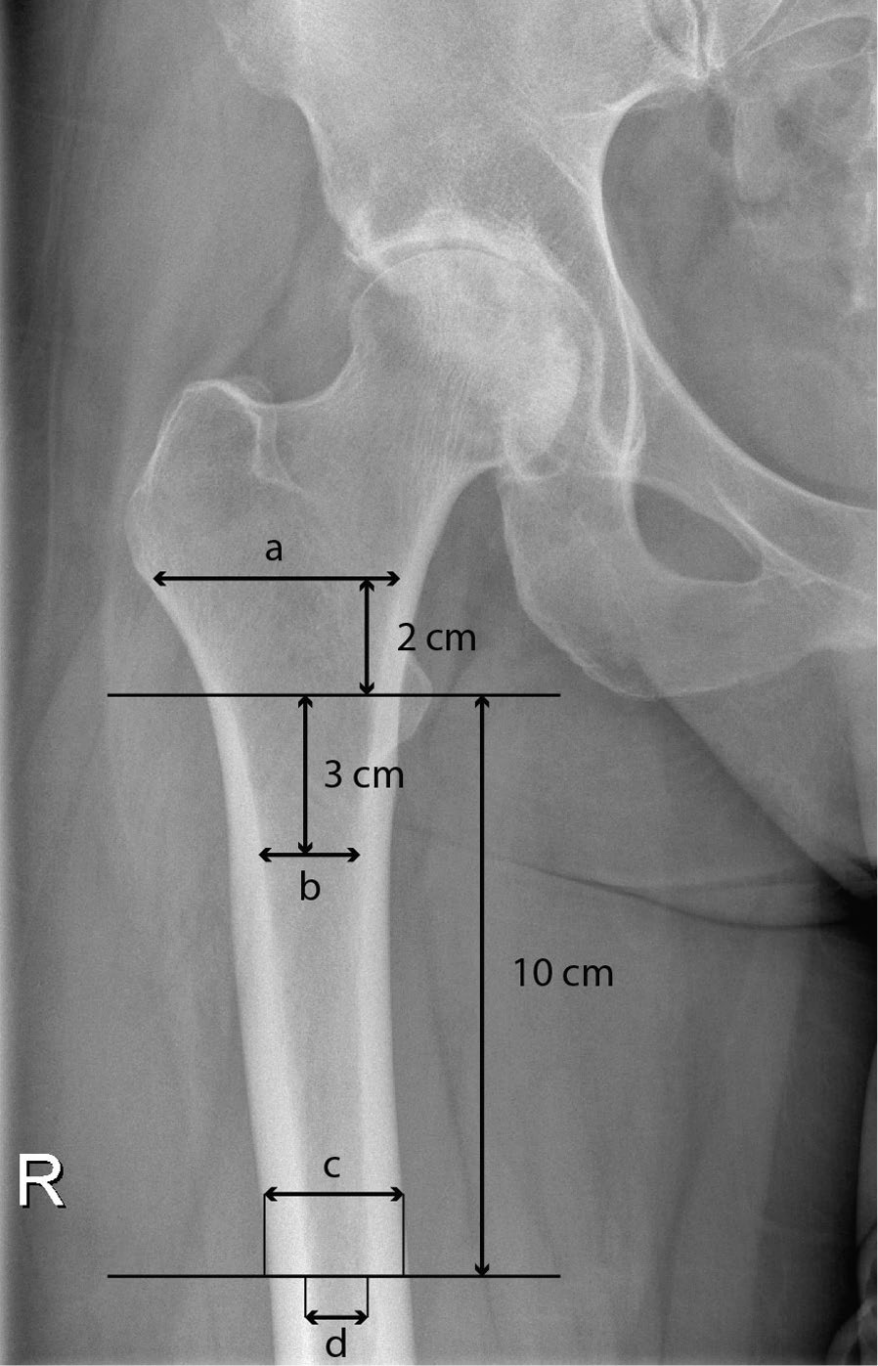
Radiological measures of proximal femur. **a** Canal width 2 cm above the mid-lesser trochanter line. **b** Canal width 3 cm below the mid-lesser trochanter line. **c** Femoral bone width 10 cm below the mid-lesser trochanter line. **d** Canal width 10 cm below the mid-lesser trochanter line. These measures were used to calculate canal flare index (*a*/*d*), cortical index [(*c* − *d*)/*c*], and canal–calcar ratio (*d*/*b*)

Radiological evaluation was carried out on standard preoperative and postoperative (on the second postoperative day) anteroposterior radiographs. A marker was used to quantify the magnification of all radiographs. Proximal femoral morphology was assessed using canal flare index (CFI), cortical index, and canal–calcar ratio (Fig. 1) [10, 11]. CFI < 3 was classified as stovepipe, CFI 3–4.7 as normal, and CFI > 4.7 as champagne flute-shaped canal [11]. In addition, the angle of the femoral component and leg length discrepancy were measured from the postoperative radiograph. Radiological evaluation was carried out by two independent raters, and the average value used for analysis. Intraclass correlation coefficient was used to assess consistency between raters.

### Statistical analysis

Statistical analysis was performed using SPSS (version 20.0 for Windows) statistical software. Data are presented as mean ± standard deviation or (range). The Student *t* test or Mann–Whitney *U* test was used to compare groups as appropriate. Chi-squared and Fisher’s exact tests were used to compare group proportions. Multivariable logistic regression analysis was used to determine independent predictors of IFF. Variables for the regression model were selected from our hypotheses and from previous studies. *p*-value of 0.05 was used as the cutoff for statistical significance.

## Results

This study included the first 222 consecutive total hip arthroplasties carried out with the new cementless SP-CL^®^ between 2015 and 2018 at Tartu University Hospital. The operations were performed on 209 patients, of whom 13 were operated bilaterally in a staged procedure. The general parameters of the study participants and the reason for arthroplasty are presented in Table 1. The mean age of study participants was 56 years (14–77 years), and their mean body mass index (BMI) was 29.6 ± 4.90 kg/m^2^, being 29.1 ± 6.2 kg/m^2^ in the IFF group versus 30.0 ± 4.9 kg/m^2^ in the nonfracture group (*p* = 0.38). The patients were operated by nine different orthopedic surgeons [five with over 5 years of experience with arthroplasty (> 100 operations/year) and four with less than 5 years of experience]. The number of operations performed using the SP-CL^®^ implant per surgeon varied from 1 to 66 with an average of 22.

**Table 1:** General characteristics of hip arthroplasty patients

Age (years)	56 (14–77)
Male/female (*n*)	112/110
Height (m)	1.71
Weight (kg)	87
BMI (kg/m^2^)	29.6 ± 4.90
Smoking (*n*, %)	
Current smoker	55 (25)
Nonsmoker	167 (75)
Diagnosis (*n*, %)	
Primary osteoarthritis	200 (90.1)
Femoral neck fracture	2 (0.9)
Posttraumatic osteoarthritis	5 (2.3)
Legg–Calve–Perthes	3 (1.4)
DDH	5 (2.3)
Idiopathic AVN	6 (2.7)
Revision (MoM acetabular component loosening)	1 (0,5)
Comorbidities (*n*)	1.2 ± 1
Length of hospital stay (days)	5.4 ± 1.4
Surgical approach (*n*, %)	
Direct lateral	116 (52)
Posterolateral	106 (48)

*BMI* body-mass index, *DDH* developmental dysplasia of hip, *AVN* avascular necrosis, *MoM* metal-on-metal

**Fig. 2:**
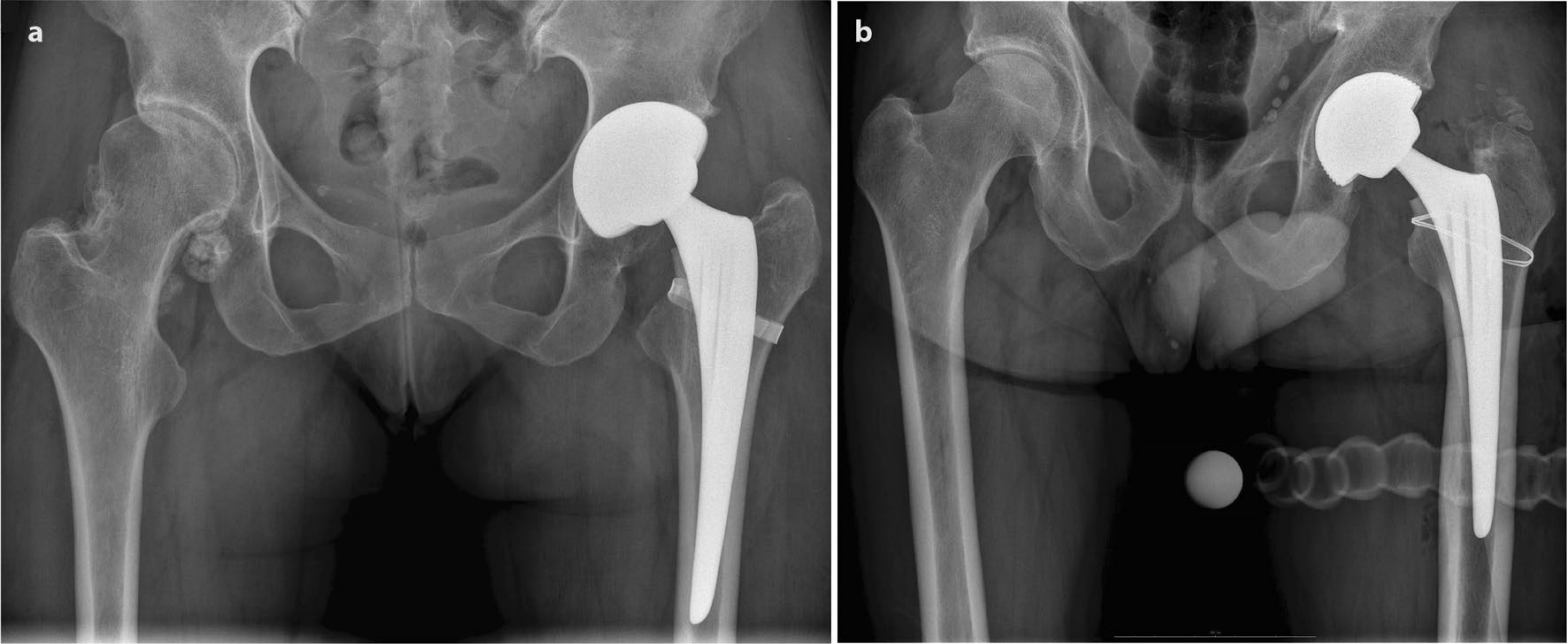
**a** Postoperative radiograph of intraoperative femoral fracture (IFF) on the right treated with titanium band. **b** Radiograph of patient with IFF treated with cerclage wire

There were 11 (5.0%) IFF among the 222 THA. The type and treatment of the IFF are presented in Table 2. There were 7 calcar fractures, 2 lateral cortex fractures, and 2 greater trochanter fractures. Seven IFF were treated with either cerclage wire or titanium band. Examples of IFF treatment methods are presented in Fig. 2. Of the fractures, four were treated conservatively by limiting weight-bearing. Routine progressive physical therapy was performed by qualified physical therapists throughout the hospital stay, and the exercises were continued by the patients independently at home. The physical therapy included strengthening exercises for both legs (hip and knee extensors, abductors, and plantar flexors), balance, and coordination exercises. All patients were advised to use walking aids for the first 6–8 weeks. The average hospital stay was not significantly different in the IFF compared with the no-fracture group (5.9 ± 1.5 versus 5.3 ± 1.3 days, *p* = 0.18, respectively).

**Table 2:** Intraoperative fracture type and treatment

Fracture type	*N*	Treatment
Subtrochanteric (lateral cortex)	2	Conservative
Greater trochanter fracture	2	Cerclage wire
Calcar	7	Titanium band (3)
		Cerclage wire (2)
		Conservative (2)

**Fig. 3:**
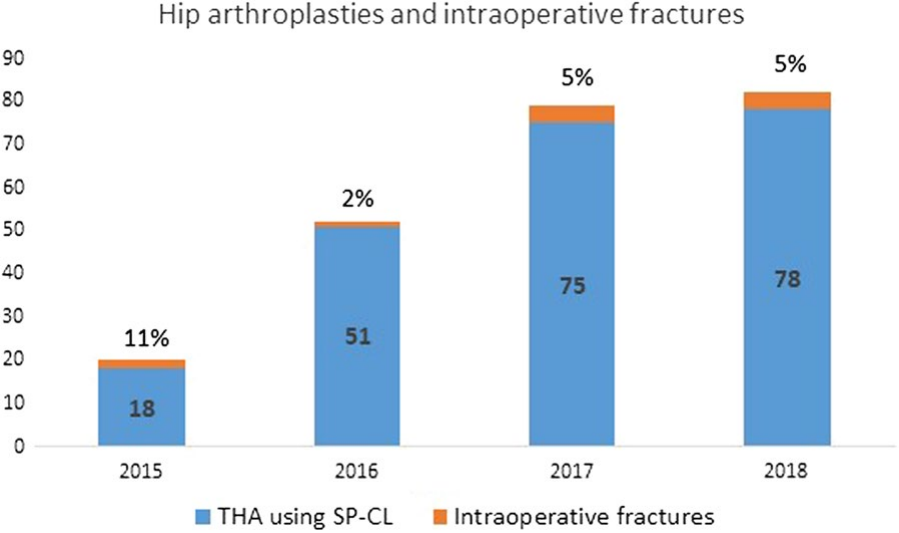
Total hip arthroplasties (THA) using SP-CL implant per year and proportion of intraoperative fractures

One of the IFF patients was revised 1 month after primary THA due to infection. All patients were invited to routine follow-up after 6 months and 1 year after surgery, where clinical (complaints, range of motion, and satisfaction) and radiological (radiolucent zones and migration of the implant) parameters were assessed by the physician. The mean follow-up for IFF patients was 17 months (3–34 months). None of the IFF patients were revised due to IFF during the follow-up period. Surgical approach, age, gender, and implant size were not different between the THA patients with and without IFF (Table 3). The number of operations and proportion of IFF per year are presented in Fig. 3.

**Table 3:** Radiological and clinical characteristics of fracture and no-fracture groups

Variable	Fracture group	No-fracture group	*p*
Age (years)	57 ± 12	56 ± 8.3	0.53
BMI (kg/m^2^)	29.1 ± 6.2	30.0 ± 4.9	0.38
Cortical index	0.59 ± 0.04	0.57 ± 0.06	0.65
Canal–calcar ratio	0.62 ± 0.09	0.66 ± 0.09	0.11
CFI according to Noble			0.02
Stovepipe (*n*, %)	6 (55)	40 (19)	
Normal (*n*, %)	5 (45)	163 (77)	
Champagne flute (*n*, %)	0 (0)	8 (4)	
Leg length discrepancy (mm)	3 ± 13	7 ± 7	0.83
Surgical approach			0.54
Direct lateral (*n*, %)	4 (36)	102 (48)	
Posterolateral (*n*, %)	7 (64)	109 (52)	
Comorbidities (*n*, range)	1.3 (0-3)	1.2 (0-6)	0.68

*BMI* body mass index, *CFI* canal flare index

In addition, there was 1 undisplaced acetabular fracture and 1 sciatic nerve injury, both of which were treated conservatively and did not cause any long-term disability, according to clinical records.

### Radiological measurements

The results of the radiographic anatomy measurements are presented in Table 3. There were no statistically significant differences regarding the canal–calcar ratio, cortical thickness index, or leg length discrepancy between the patients with versus without fracture (Table 3). However, the proportion of patients with stovepipe-shaped proximal femur was higher in the fracture compared with no-fracture group.

In the logistic regression analysis, proximal femur CFI shape and canal–calcar ratio were significant predictors of IFF (Table 4). Other potential risk factors were included in the analysis and did not show a statistically significant impact.

**Table 4:** Binary logistic regression model for intraoperative fractures using SP-CL implant

Variable	OR	95% CI	*p*
CFI shape	0.13	0.03–0.55	0.006
Canal–calcar ratio	< 0.01	0.00–0.38	0.032
Surgical approach	0.14	0.71–12.04	0.138
Year of operation	0.63	0.32–1.23	0.174
Gender	0.54	0.14–2.12	0.379
Age	1.02	0.94–1.11	0.599
Implant size	1.35	0.30–6.05	0.696

*CFI* canal flare index

CFI shape was stovepipe, normal, or champagne flute configuration; surgical approach coded as 1 for direct lateral and 2 for posterolateral; Gender coded as 1 for male and 0 for female; implant sizes split at the median value and classified as 0 for smaller and 1 for larger size

Consistency among the raters was assessed with ICC. The ICC for CFI was excellent (0.92, CI 0.89–0.94) or good for cortical thickness index (0.77, CI 0.70–0.82) and moderate for canal–calcar ratio and leg length discrepancy (0.74, CI 0.66–0.80 and 0.63, CI 0.52–0.72, respectively).

## Discussion

This is the first study to evaluate intraoperative complications associated with THA using the new cementless femoral implant SP-CL^®^. We found that, in the first consecutive 222 THA, the most prevalent intraoperative complication was femoral fracture with incidence of 5%. The IFF risk was associated with the radiological anatomy of the proximal femur.

The rate of IFF in THA using cementless implants generally ranges between 0.6% and 6%, although incidence above 20% has also been reported [8, 12–14, 16]. As IFF increases reoperation risk and causes poor functional outcome, it is crucial to minimize modifiable risk factors and choose the correct implant for each patient [9, 15]. Previously, the radiological shape of the proximal femur has been associated with IFF risk [16]. In support of this, we found that classification according to Noble CFI into either stovepipe, normal, or champagne flute configuration was an independent predictor of IFF (Table 4). In addition, canal–calcar ratio also predicted IFF. As these markers have previously been associated with bone mineral density, the results of our study suggest that poor bone quality may increase IFF risk [17, 18].

Several clinical risk factors have been reported for IFF of THA: female gender, greater age, implant size, anterior and lateral (Hardinge) approach, and obesity [14, 16]. Age was not an independent predictor of IFF in our study. This might be associated with the significantly lower average age of the study participants compared with previous studies [16, 19]. There was no significant difference in fracture rate regarding the surgical approach in our study (Table 3). Several studies have found an increased risk in the case of direct lateral and anterolateral approach; however, different type of implants were used in those studies, which might at least partly explain the different results [16, 20]. Furthermore, in our study, obesity, gender, and implant size were not independently associated with IFF. This might be due to the relatively small sample size and the low incidence of IFF. Also, mastering the specific technical features of a new implant takes time and has a learning curve that might have influenced the results. The complexity of measuring the impact of the learning curve has been acknowledged before [21]. In the current study, we analyzed the effect of operation year and operating surgeon (both individually and categorized into two groups by experience) and found no significant impact of these variables on logistic regression analysis.

Miettinen et al. analyzed THA complications and found a 3.7% incidence rate of IFF [16]. Their analysis provides a good comparison with the current study because it was done in the same geographical region, most (81%) of the implants used were of fit-and-fill design (relatively similar to SP-CL^®^), and the age and gender proportions of the patients are similar. The IFF rate was comparable with the current study, and they also found that the geometry of the proximal femur was an independent risk factor for IFF.

Cementless femoral implants for THA have different stem designs and contact areas between the bone and implant. Therefore, the properties of primary and secondary fixation vary. Because of the growing number of different femoral stems used in clinical practice, there is no comprehensive classification system covering all implants. According to the Mount group classification, SP-CL^®^ is the closest to type 6 with an anatomic shape and tapered conical design to provide maximal fit and fill in the metaphyseal area [22]. The SP-CL^®^ used in the current study is made of titanium alloy, which is one of the most prevalent materials used in cementless femoral components due to its various advantages over stainless-steel and cobalt-chromium alloys. The stiffness of titanium implants is more similar to that of bone compared with stainless-steel alloys, and titanium exhibits better bone ingrowth properties compared with cobalt-chromium implants [23]. The use of primary cementless stems in revision surgery is controversial, and only a few studies can be found [24]. Nevertheless, if the bone quality is good and there is no substantial osteolysis, then the use of a primary cementless stem can be a good option. Using a primary cementless stem in revision arthroplasty can prevent treatment escalation without limiting future implant selection and has been encouraged previously by some authors [24, 25].

Bone stress shielding is a well-established issue with cementless THA, especially in the younger population, and is associated with thigh pain [26]. Anatomic shape, elastic properties, and grooves in the proximal side of SP-CL^®^ have been shown to provide better results in strain shielding compared with other implants in finite-element analysis [27]. The implant choice depends on many things and should always be based on the patient’s individual characteristics, taking into account the anatomical shape and proportions of the proximal femur to identify the best-fitting implant. Also, bone quality, which can be assessed using the Dorr and Noble classification on radiographs, should be taken into account [10, 11].

Several technical aspects of the operation have been noted by the authors and should be taken into consideration when using the SP-CL^®^ implant: the femoral neck cut must be sufficiently low to avoid creation of a high-stress region when preparing for and inserting the implant. On the other hand, too high a femoral neck cut can lead to undersizing and malpositioning (varus) of the implant. To minimize the risk of fracture during implantation, the SP-CL^®^ should be inserted using a number of low-energy strikes until it cannot be pushed any deeper into the canal.

Acetabular fracture is uncommon in THA but if missed during surgery can lead to early reoperation and poor clinical results [28]. There was one intraoperative acetabular fracture that was diagnosed on the postoperative radiograph and managed conservatively with limited weight-bearing. Acetabular fractures are associated with cementless implants and occur most often during the insertion of the acetabular cup. It is hard for the surgeon to control the impaction strike precisely. High-energy strikes lead to a marginal increase in the pushout fixation but only a small decrease in the polar gap and thus should be avoided [29].

Nerve injury during THA is a rare complication but can have disastrous consequences [30]. In the current study, there was one patient who had a sciatic nerve injury with foot drop postoperatively, which was resolved with conservative management. The sciatic nerve is more at risk when the posterolateral approach is used, but for this patient the direct lateral surgical approach was employed. The transitory nerve injury probably occurred during the preparation of the femoral canal when the retractors were used to make way for the canal compactors and pressed on the nerve.

The strengths of this study include its comprehensive assessment of the clinical and radiological risk factors for IFF and the fact that all first consecutive arthroplasties using the SP-CL^®^ carried out at Tartu University Hospital were included in the study, thus minimizing any bias. However, it should be noted that the sample size was relatively small and the rate of IFF was low, meaning that the study lacks statistical power to identify independent risk factors. The results of the logistic regression analysis should thus be interpreted with caution. Furthermore, its retrospective design does not allow the confirmation of causal relationships. The operations were performed by nine different orthopedic surgeons with different experience in THA. Also, since Tartu University Hospital is a teaching hospital, residents are involved in surgeries, which might have an impact on the complication rate and must be accounted for when extrapolating the results of the study. In addition, the absence of a comparison group in which a different implant design was used by the same surgeons is another limitation of the study.

In conclusion, intraoperative complications related to the use of the new femoral implant SP-CL^®^ in THA are described herein. Iatrogenic IFF were the most common complications, with incidence of 5%. The radiological morphology of the proximal femur was an important predictor of IFF and should be assessed when the SP-CL^®^ is used. None of the IFF patients required revision due to the fracture. Cementless implants should be avoided in patients with poor bone quality because of higher complication risk. Further research is needed to assess the mid- and long-term results of the SP-CL^®^ in comparison with other cementless implants in THA.

## Data Availability

The datasets used and analyzed during the currents study are available from the corresponding author on reasonable request.

## References

[1] Learmonth ID, Young C, Rorabeck C (2007). The operation of the century: total hip replacement. Lancet.

[2] Bayliss LE, Culliford D, Monk AP, Glyn-Jones S, Prieto-Alhambra D, Judge A (2017). The effect of patient age at intervention on risk of implant revision after total replacement of the hip or knee: a population-based cohort study. Lancet.

[3] Kurtz SM, Lau E, Ong K, Zhao K, Kelly M, Bozic KJ (2009). Future young patient demand for primary and revision joint replacement: national projections from 2010 to 2030. Clin Orthop.

[4] Chidambaram R, Cobb A (2009). Change in the age distribution of patients undergoing primary hip and knee replacements over 13 years—an increase in the number of younger men having hip surgery. Orthop Proc.

[5] Ulrich SD, Seyler TM, Bennett D, Delanois RE, Saleh KJ, Thongtrangan I (2008). Total hip arthroplasties: what are the reasons for revision?. Int Orthop.

[6] Wechter J, Comfort TK, Tatman P, Mehle S, Gioe TJ (2013). Improved survival of uncemented versus cemented femoral stems in patients aged < 70 years in a community total joint registry. Clin Orthop.

[7] Mäkelä KT, Matilainen M, Pulkkinen P, Fenstad AM, Havelin L, Engesaeter L (2014). Failure rate of cemented and uncemented total hip replacements: register study of combined Nordic database of four nations. BMJ.

[8] Lamb JN, Matharu GS, Redmond A, Judge A, West RM, Pandit HG (2019). Risk factors for intraoperative periprosthetic femoral fractures during primary total hip arthroplasty an analysis from the national joint registry for England and Wales and the Isle of Man. J Arthroplasty.

[9] Ferbert T, Jaber A, Gress N, Schmidmaier G, Gotterbarm T, Merle C (2019). Impact of intraoperative femoral fractures in primary hip arthroplasty: a comparative study with a mid-term follow-up. Hip Int J Clin Exp Res Hip Pathol Ther.

[10] Dorr LD, Faugere MC, Mackel AM, Gruen TA, Bognar B, Malluche HH (1993). Structural and cellular assessment of bone quality of proximal femur. Bone.

[11] Noble PC, Alexander JW, Lindahl LJ, Yew DT, Granberry WM, Tullos HS (1988). The anatomic basis of femoral component design. Clin Orthop.

[12] Abdel MP, Watts CD, Houdek MT, Lewallen DG, Berry DJ (2016). Epidemiology of periprosthetic fracture of the femur in 32,644 primary total hip arthroplasties. Bone Jt J.

[13] Stuchin SA (1990). Femoral shaft fracture in porous and press-fit total hip arthroplasty. Orthop Rev.

[14] Ponzio DY, Shahi A, Park AG, Purtill JJ (2015). Intraoperative proximal femoral fracture in primary cementless total hip arthroplasty. J Arthroplasty.

[15] Thillemann TM, Pedersen AB, Johnsen SP, Søballe K (2008). Inferior outcome after intraoperative femoral fracture in total hip arthroplasty: outcome in 519 patients from the Danish Hip Arthroplasty Registry. Acta Orthop.

[16] Miettinen SSA, Mäkinen TJ, Kostensalo I, Mäkelä K, Huhtala H, Kettunen JS (2016). Risk factors for intraoperative calcar fracture in cementless total hip arthroplasty. Acta Orthop.

[17] Yeung Y, Chiu KY, Yau WP, Tang WM, Cheung WY, Ng TP (2006). Assessment of the proximal femoral morphology using plain radiograph-can it predict the bone quality?. J Arthroplasty.

[18] Sah AP, Thornhill TS, LeBoff MS, Glowacki J (2007). Correlation of plain radiographic indices of the hip with quantitative bone mineral density. Osteoporos Int J Establ Result Coop Eur Found Osteoporos Natl Osteoporos Found USA.

[19] Hartford JM, Knowles SB (2016). Risk factors for perioperative femoral fractures: cementless femoral implants and the direct anterior approach using a fracture table. J Arthroplasty.

[20] Zhao R, Cai H, Liu Y, Tian H, Zhang K, Liu Z (2017). Risk factors for intraoperative proximal femoral fracture during primary cementless THA. Orthopedics.

[21] Khan N, Abboudi H, Khan MS, Dasgupta P, Ahmed K (2014). Measuring the surgical “learning curve”: methods, variables and competency. BJU Int.

[22] Kim JT, Yoo JJ (2016). Implant design in cementless hip arthroplasty. Hip Pelvis.

[23] Matassi F, Botti A, Sirleo L, Carulli C, Innocenti M (2013). Porous metal for orthopedics implants. Clin Cases Miner Bone Metab.

[24] Gastaud O, Cambas PM, Tabutin J (2016). Femoral revision with a primary cementless stem. Orthop Traumatol Surg Res.

[25] Cavagnaro L, Formica M, Basso M, Zanirato A, Divano S, Felli L (2018). Femoral revision with primary cementless stems: a systematic review of the literature. Musculoskelet Surg.

[26] Taylor WR, Szwedowski TD, Heller MO, Perka C, Matziolis G, Müller M (2012). The difference between stretching and splitting muscle trauma during THA seems not to play a dominant role in influencing periprosthetic BMD changes. Clin Biomech Bristol Avon.

[27] Heyland M, Checa S, Kendoff D, Duda GN (2019). Anatomic grooved stem mitigates strain shielding compared to established total hip arthroplasty stem designs in finite-element models. Sci Rep.

[28] Haidukewych GJ, Jacofsky DJ, Hanssen AD, Lewallen DG (2006). Intraoperative fractures of the acetabulum during primary total hip arthroplasty. J Bone Joint Surg Am..

[29] Doyle R, van Arkel RJ, Jeffers JRT (2019). Effect of impaction energy on dynamic bone strains, fixation strength, and seating of cementless acetabular cups. J Orthop Res Off Publ Orthop Res Soc.

[30] Hasija R, Kelly JJ, Shah NV, Newman JM, Chan JJ, Robinson J (2018). Nerve injuries associated with total hip arthroplasty. J Clin Orthop Trauma.

